# Implications of MMP9 for Blood Brain Barrier Disruption and Hemorrhagic Transformation Following Ischemic Stroke

**DOI:** 10.3389/fncel.2016.00056

**Published:** 2016-03-04

**Authors:** Renée J. Turner, Frank R. Sharp

**Affiliations:** ^1^Discipline of Anatomy and Pathology, Adelaide Centre for Neuroscience Research, School of Medicine, The University of AdelaideAdelaide, SA, Australia; ^2^Department of Neurology, MIND Institute, University of California at Davis Medical CenterSacramento, CA, USA

**Keywords:** neutrophils, MMP-9, tPA, blood-brain barrier, ischemic stroke, hemorrhagic transformation, cerebral edema

## Abstract

Numerous studies have documented increases in matrix metalloproteinases (MMPs), specifically MMP-9 levels following stroke, with such perturbations associated with disruption of the blood brain barrier (BBB), increased risk of hemorrhagic complications, and worsened outcome. Despite this, controversy remains as to which cells release MMP-9 at the normal and pathological BBB, with even less clarity in the context of stroke. This may be further complicated by the influence of tissue plasminogen activator (tPA) treatment. The aim of the present review is to examine the relationship between neutrophils, MMP-9 and tPA following ischemic stroke to elucidate which cells are responsible for the increases in MMP-9 and resultant barrier changes and hemorrhage observed following stroke.

## Introduction

Over the last decade the matrix metalloproteinases (MMPs) have been widely investigated for their role in disruption of the blood-brain barrier (BBB), particularly the extracellular matrix (ECM), following stroke (Romanic et al., 1998; Rosenberg et al., 1998; Fujimura et al., 1999; Gasche et al., 1999; Gidday et al., 2005) and other cerebral pathologies such as traumatic brain injury (Planas et al., 2001) and neoplasm (Lukes et al., 1999; Turba et al., 2007). MMPs are a family of zinc and calcium-dependent endopeptidases that are capable of degrading all components of the ECM including laminin, collagen and fibronectin, amongst many other targets (Van den Steen et al., 2002). At least 23 MMPs have been identified to date (Sternlicht and Werb, 2001), with MMP-2 and MMP-9 the most widely studied in stroke. In particular, MMP-9 has been implicated, not only in the pathogenesis of BBB breakdown and subsequent vasogenic edema formation following stroke (Fujimura et al., 1999; Gasche et al., 1999; Rosenberg and Yang, 2007), but also in hemorrhagic transformation (HT) in the setting of tissue plasminogen activator (tPA) therapy (Lapchak et al., 2000; Wang et al., 2009). Cerebral edema and HT of the infarct are significant problems in clinical stroke, which are associated with poor outcome and contribute to the morbidity and mortality of this condition (Hacke et al., 1996; Fiorelli et al., 1999). Elucidating the mechanisms of such deleterious events is the key to developing targeted, more effective clinical therapies.

Numerous clinical and experimental studies have confirmed an increase in serum MMP-9 following stroke (Clark et al., 1997; Romanic et al., 1998; Yushchenko et al., 2000; Montaner et al., 2003a; Ning et al., 2006). However, the cellular source of this MMP-9 remains controversial. Although it is generally accepted that MMP-9 is increased following stroke, there is debate as to which cells are responsible, whether it be resident brain cells, cells of the vasculature or circulating immune cells, such as neutrophils. However, the aim of the present review was to explore the potential relationship between neutrophil-derived MMP-9 and complications such as BBB disruption and HT following stroke to elucidate the cellular source of MMP-9 in ischemic stroke.

### Matrix metalloproteinases

MMPs regulate many aspects of cellular activity with functions ranging from ECM degradation, cell proliferation, adhesion, and migration to release of ECM-sequestered molecules by proteolysis, shedding of cell-surface proteins that transduce signals from the ECM (Cunningham et al., 2005) and activation of pro-inflammatory cytokines (Candelario-Jalil et al., 2009). As such, recognized targets of MMP-9 include components of the ECM, tight junction components, growth factors and their precursors, cell surface receptors and cell adhesion molecules (Bajor and Kaczmarek, 2013; Vandooren et al., 2013; Conant et al., 2015). The MMPs may have pleiotropic actions on target tissues, with MMPs integrally involved in the normal remodeling of tissue during development and homeostasis but dysregulation of MMPs is implicated in disease states and has repercussions for BBB integrity, tissue injury and cell death (Agrawal et al., 2008). However, action of the MMPs, including MMP-9, have been well documented to play critical roles in tissue repair and remodeling following stroke (Lenglet et al., 2015), particularly in angiogenesis and re-establishment of cerebral blood flow with long-term MMP inhibition shown to markedly reduce neuronal plasticity and impair vascular remodeling (Zhao et al., 2006, 2007).

Given that uncontrolled expression of MMPs can result in tissue injury and destruction, the catalytic activity of MMPs is regulated at four points, which are: gene expression level, compartmentalization of the MMPs, pro-enzyme activation, and enzyme inactivation (Ra and Parks, 2007). Cleavage of the prop-peptide renders the MMP proteolytically active. However, given that one cysteine residue in the pro-peptide domain coordinates the catalytic site, disruption of this site via S-nitrosylation can also activate MMP-9 (Gu et al., 2002; Manabe et al., 2005; McCarthy et al., 2008). MMP is activity is further controlled by the availability and affinity of substrates. Indeed, MMPs are normally expressed at very low levels under normal conditions with localized expression induced when remodeling of the ECM is required. An in depth discussion of the transcription and regulation of MMPs is beyond the scope of the present review; we refer readers to some excellent reviews on the control of MMP activity (Ra and Parks, 2007; Clark et al., 2008; Fanjul-Fernández et al., 2010). Furthermore, MMPs are tightly regulated at both the transcriptional and post-transcriptional level by transcription factors and inhibitor proteins (Clark et al., 2008). In particular, endogenous tissue inhibitors of metalloproteinases (TIMPs), through high affinity non-covalent binding to the MMP catalytic domain, inhibit the activity of MMPs. To date, four TIMPs have been identified, with TIMP-1 having a specific affinity for MMP-9 (Clark et al., 2008; Fujimoto et al., 2008). In addition, MMPs, such as MMP-9, are secreted as inactive zymogens (proforms) that require activation through cleavage of the pro-peptide. This cleavage produces a conformational change, enabling a water molecule to associate with MMP-9, rendering it proteolytically active (Clark et al., 2008), and thereby providing another level of control for MMP function. In particular, pro-MMP-9 may be activated by a number of molecules including MMP-2, MMP-3, plasmin, urokinase-type plasminogen activator (uPA), and tPA (Rosenberg et al., 2001; Cunningham et al., 2005). Indeed, another level of control is conferred by the influence of growth factors, cytokines, and chemokines on both MMP and TIMP transcription (Yan and Boyd, 2007), with a response at the transcriptional level typically occurring within a few hours of stimulation.

Certainly MMP-9 has been the most widely studied MMP family member in both the experimental and clinical stroke literature, which may in part be due to the fact that it can easily be assessed using techniques such as gelatin zymography. Nevertheless, the advancement of other techniques such as multiplex ELISAs and proteome arrays has allowed the identification and quantification of the role of other MMP family members following stroke and their contribution to tissue injury.

## MMP-9 in experimental ischemic stroke

Increased expression of pro/active MMP-9 has been detected within hours to days following stroke in non-human primates (Heo et al., 1999), rats (Romanic et al., 1998; Rosenberg et al., 1998; Justicia et al., 2003), and mice (Fujimura et al., 1999; Gasche et al., 1999; Asahi et al., 2001a). Following stroke, increased levels of MMP-9 have been detected in both peripheral and central cells including neurons, glia, endothelial cells and neutrophils, with each of these cell types having a unique MMP secretion/expression profile (Rosenberg, 2002; Van den Steen et al., 2002; Gasche et al., 2006). However, the expression of MMPs is highly dependent upon the type, duration and severity of the ischemic insult, in addition to the animal species and strain used, with the temporal profile of MMP expression varying widely amongst studies. Another level of complexity in comparison of studies is that both pro-MMP and active MMP levels are not always reported in concert. Altogether, these issues highlight the complexity in targeting this protease with treatment following ischemic stroke.

Indeed, alterations in other MMPs beyond MMP-9 have been observed following stroke. For example, increased levels of pro/active MMPs including MMP-2 (Amantea et al., 2008; Lenglet et al., 2014), MMP-3 (Kim et al., 2005; Si-Tayeb et al., 2006; Yang et al., 2011; Lenglet et al., 2014), MMP-4 (Lenglet et al., 2014), MMP-10 (Lenglet et al., 2014), and MMP-13 (Cuadrado et al., 2009). Such alterations in MMPs (and their endogenous inhibitors) has been linked to activation of astrocytes and microglia (Kim et al., 2005; Yang et al., 2011), increased levels of circulating inflammatory cytokines (Amantea et al., 2014) and enhanced thrombolysis (Orbe et al., 2011).

### Blood-brain barrier breakdown

Disruption of the BBB is a key event in the secondary injury cascade following stroke, one that exacerbates injury through a number of mechanisms including permitting the entry of peripheral immune cells into the brain to enhance the neuroinflammatory response, and hasten the development of vasogenic edema (Kuroiwa et al., 1985). Given their ability to degrade the ECM and tight junction components, MMPs have been implicated in BBB permeability alterations post-stroke.

Following stroke, a biphasic opening of the BBB is well established (Kuroiwa et al., 1985). The first alteration in BBB permeability occurs within hours of stroke onset, with the second occurring some 24–48 h later (Rosenberg et al., 1998). Such early and late alterations in barrier permeability are consistent with the increased expression of MMP-2 and MMP-9, which are the main MMPs that have been shown to be altered following both stroke and traumatic brain injury (Mun-Bryce and Rosenberg, 1998; Romanic et al., 1998; Fujimura et al., 1999; Gasche et al., 1999; Asahi et al., 2000; Planas et al., 2001; Rosenberg et al., 2001). Specifically, in one study increased levels of MMP-2 have been observed, in concert with an early and reversible disruption to the BBB (Chang et al., 2003), with late BBB disruption at 24–48 h following stroke observed in conjunction with increased MMP-9 levels (Sandoval and Witt, 2008). The early BBB disruption attributable to increased MMP-2 levels is deemed reversible, as although the tight junction components loosen, they remain within the endothelial cleft, and thus can be reassembled to reverse such permeability changes (Yang et al., 2007). In contrast, the delayed breach in BBB integrity in the setting of elevated MMP-9 expression is associated with complete degradation of the basal lamina (Mun-Bryce and Rosenberg, 1998) and tight junction components (Asahi et al., 2001b) resulting in and gross barrier disruption. This late barrier disruption persists for several days and is associated with complete breakdown of the BBB and HT (Romanic et al., 1998; Rosenberg et al., 1998; Asahi et al., 2001b). Due to the integral function of the BBB in the development of cerebral oedema, changes in MMP-9 levels have also been shown to correlate with the severity of cerebral edema, in a rodent model of stroke (Li et al., 2013). This scenario of early MMP2 and late MMP9, however, has been challenged by a number of other studies described below that implicate neutrophil MMP9 in early BBB opening (Montaner et al., 2008).

In addition, despite the involvement of MMP-2 in early barrier disruption, MMP-2 inhibition does not confer protection against BBB disruption (Asahi et al., 2001b; Gidday et al., 2005). Indeed, administration of the specific MMP-2/9 inhibitor SB-3CT failed to confer protection against a hypoxic-ischemic insult in neonatal rats (Ranasinghe et al., 2012). In contrast, MMP-9 inhibition provides robust protection against changes in BBB permeability (Svedin et al., 2007), suggesting that MMP-9 is the dominant protease acting at the BBB following ischemic stroke (Dejonckheere et al., 2011). MMP-3 immunoreactivity has been observed in pericytes following stroke and given that MMP-3 activates MMP-9 *in vivo*, MMP-3 knockout mice have been investigated to explore the effects of MMP-9 in ischemic stroke (Gurney et al., 2006). MMP-3 knockout reduced the levels of active MMP-9 and subsequent BBB disruption and was associated with a significantly reduced number of neutrophils infiltrating the stroke lesion.

Oxidative stress is known to play a significant role in the evolution of injury following stroke. Through studies in superoxide dismutase (SOD) 1-knockout mice, a role for oxidative stress in the mediation of BBB disruption has been revealed (Gasche et al., 2001). Specifically, higher levels of pro-MMP-9 and active MMP-9, in conjunction with profound BBB disruption were observed in SOD1 knockouts, an effect reversed with MMP inhibition, with comparable findings also reported in SOD2 knockouts (Maier et al., 2004). Subsequent studies have revealed nitric oxide and ROS to be the specific components of the oxidative stress response that lead to MMP-9 activation (Gu et al., 2002), validated by the significant decrease in infarct volume, vascular damage and MMP-9 activation following treatment with a non-selective nitric oxide inhibitor (Gürsoy-Ozdemir et al., 2000).

### Hemorrhagic complications

As many as 88% of strokes are ischemic in type and therefore may benefit from thrombolysis with tPA to recanalize the occluded cerebral artery (Adibhatla and Hatcher, 2008). In addition to its actions as a thrombolytic agent, tPA, via activation of MMP-9, may also damage the basal lamina and tight junctions of the cerebral blood vessels, resulting in increased permeability of the BBB, cerebral edema, and hemorrhagic complications (Lapchak et al., 2000; Sumii and Lo, 2002). MMP-induced degradation of the ECM is problematic as it weakens vessels, making them more prone to rupture and increases risk of cerebral hemorrhage (Heo et al., 1999; Lapchak et al., 2000; Montaner et al., 2001a; Sumii and Lo, 2002; Rosenberg and Yang, 2007; Rosell et al., 2008). Though a number of proteases may activate MMP-9, plasmin and tPA are two of the most important in the setting of stroke as they have implications in hemorrhagic complications. Indeed, such secondary disturbances to the BBB and microvascular damage precede such HT. Thrombolysis-induced hemorrhage is classified into two main types: PH and HT. In PH there is a discrete loss of microvascular integrity and extravasation of red blood cells into the brain parenchyma, whereas HT involves gross disruption to the cerebral vasculature and hematoma development. However, it must be noted that tPA-induced activation of MMP-9 may be beneficial in the late reparative phase of stroke to assist in the vascular remodeling, angiogenesis, neurogenesis and axonal regeneration response.

In addition to driving BBB disruption, MMP-9 has also been implicated in HT following ischemic stroke. Treatment with tissue-type tPA increases MMP-9 levels after embolic stroke in rodents, thereby implicating MMPs in tPA-induced hemorrhage (Sumii and Lo, 2002). Furthermore, MMP inhibitors have been shown to reduce the incidence and severity of tPA-induced hemorrhagic complications (Lapchak et al., 2000; Sumii and Lo, 2002). Minocycline (either low dose IV or high dose IP) inhibited MMP-9 upregulation induced by tPA treatment (Machado et al., 2009) and was shown to extend the 3 h time window for tPA administration to 6 h in a embolic model of ischemic stroke in rats (Murata et al., 2008).

### MMP inhibition and knockout studies

Reduced breakdown of the BBB has been reported with broad inhibition of MMPs (Ferry et al., 1997; Fernandez-Patron et al., 2001) or targeted MMP-9 gene deletion (Asahi et al., 2000; Gasche et al., 2001; Rosenberg, 2002). Indeed, elevated active MMP-9 was observed as early as 3 h post-stroke and peaking at 18 h post-stroke onset, and thus preceded marked BBB disruption at 6 h and cerebral edema at 24 h. Increased MMP-9 levels were involved in such events as MMP inhibition with GM6001 ameliorated BBB permeability changes and cerebral edema (Shigemori et al., 2006). Specifically, MMP-9 knockout animals have reduced infarct volumes, incidence of HT, volume of cerebral edema, and functional deficits compared to wild type animals (Romanic et al., 1998; Asahi et al., 2000, 2001b; Lee et al., 2003; Hu et al., 2009; Wang et al., 2009). Similarly, MMP inhibitor treatment reduced MMP-9 activation and attenuated disorganization of tight junction proteins, including occludin and zona-occludens-1, leading to a reduction in vascular leakage and preservation of BBB integrity (Bauer et al., 2010). Furthermore, MMP inhibition with GM6001 ameliorated BBB disruption at 6 h and cerebral edema at 24 h post-stroke (Shigemori et al., 2006). Studies administering MMP inhibitors prior to stroke have also shown benefit (Asahi et al., 2000). However, as these molecules are unable to cross the intact BBB it is likely that they are acting on inflammatory cells or endothelial cells, providing it can gain access to them. The fact that such agents are beneficial suggests that an effect on circulating leukocytes with a subsequent reduction in MMP-9 is plausible.

## MMP-9 in clinical ischemic stroke

In keeping with the experimental literature, studies of clinical stroke patients have revealed increased levels of MMP-9 following ischemic stroke in humans (Anthony et al., 1997; Montaner et al., 2003b; Ning et al., 2006; Rosell et al., 2008) with elevated MMP-9 levels observed compared to healthy controls (Lucivero et al., 2007). Gene expression studies of peripheral blood from stroke patients reveled that MMP-9 was one of the key genes upregulated in response to stroke (Tang et al., 2006). Furthermore, MMP-9 has been shown to be elevated in the serum of stroke patients and is correlated with a worsened outcome (Montaner et al., 2001b; Copin et al., 2005; Ning et al., 2006). This elevation in MMP-9 was determined to be a marker of stroke for patients arriving within 12 h of stroke onset (Reynolds et al., 2003), with MMP-9 levels even proposed as a marker that could predict the probability of stroke (Lynch et al., 2004). Indeed, elevations in other MMPs, such as MMP-10, have been observed in clinical patients following stroke. Increased serum pro-MMP-10 levels were observed in both tPA-treated and non-tPA-treated patients compared to age-matched controls. Such alterations in pro-MMP-10 were associated with large infarct volumes, development of severe brain edema, neurological deterioration, and poor outcome at 3 months but interestingly, elevations in pro-MMP10 were not associated with hemorrhagic transformation (Rodríguez et al., 2013). Such studies highlight the breadth of MMP alterations following stroke and the implications for infarct evolution and both the development and treatment of complications.

### Outcome following stroke

In ischemic stroke patients, a correlation between plasma MMP-9 levels and final National Institute of Health Stroke Scale (NIHSS) score, which is used to objectively quantify the impairment cause be stroke, has been observed (Montaner et al., 2001b). MMP-9 expression correlated with stroke severity and poor outcome, as assessed by the NIHSS (Inzitari et al., 2013) at 48 h post-stroke (Montaner et al., 2001b) but was shown to correlate with NIHSS score early as 24 h post-stroke onset (Rosell et al., 2005). Furthermore, there was a positive correlation between MMP-9 levels and NIHSS score and a negative correlation with the Barthel Index, a measure of activities of daily living (Vukasovic et al., 2006). Although a subsequent study by Montaner showed that high MMP-9 levels correlated with NIHSS score at admission this did not identify a specific stroke etiology (Montaner et al., 2008). Potential explanations for the differences in the outcomes of these studies may include the composition of the patient cohorts and variations in stroke size and type. In regards to long-term outcomes, MMP-9 was associated with a poor neurological outcome at 3 months post-stroke (Rodríguez-Yáñez et al., 2006) and hyperacute levels of MMP-9 correlated with worse Rankin outcome at 3 months post-stroke (Ning et al., 2006). This poor outcome and increased levels of MMP-9 were subsequently correlated with both infarct volume and stroke severity (Ning et al., 2006). A single study has reported that both MMP-2 and MMP-9 levels correlated with clinical severity and the extent of the infarct (Sotgiu et al., 2006).

### Infarct volume

In keeping with the role of MMP-9 in hastening BBB disruption and resultant injury, a non-significant increase in MMP-9 levels was observed in those tPA-treated patients that developed severe brain edema (Moldes et al., 2008). Furthermore, MMP-9 levels were shown to directly relate to stroke infarct volume (Horstmann et al., 2003; Rosell et al., 2005; Sotgiu et al., 2006; Vukasovic et al., 2006), a correlation that was observed at 24 h post-stroke (Rosell et al., 2005), but was apparent as early as 6 h post-stroke (Montaner et al., 2003b), with MMP-9 identified as the only marker that accurately predicted final infarct volume (Montaner et al., 2003b). As has been observed in the experimental stroke literature, MMP-9 levels increase over time following clinical stroke. MMP-9 levels in ischemic stroke patients were significantly elevated at 7d compared to 1d post-stroke (Kurzepa et al., 2006), with a temporal profile study out to 12d post-stroke revealing that levels of MMP-9 increased steadily over time following the onset of cerebral ischemia (Horstmann et al., 2003).

### Hemorrhagic complications

A relationship between baseline MMP-9 levels and late HT has been established, where high baseline levels were predictive of late hemorrhagic events (Montaner et al., 2001c), further supported by the observation that MMP-9 levels were higher in patients who developed HT of their infarct (Heo et al., 2003). Furthermore, MMP-9 levels were significantly higher in those patients with HT, compared to those without (Castellanos et al., 2003). Levels of MMP-9 even differed between those patients who developed symptomatic HT compared to non-symptomatic HT (Castellanos et al., 2003). MMP-9 levels were also a good predictor of petechial hemorrhage (PH) in tPA-treated ischemic stroke patients (Castellanos et al., 2007). Indeed, elevated MMP-9 levels were associated with increased symptomatic intracerebral hemorrhage (ICH) or death (Inzitari et al., 2013), leading to the suggestion that MMP inhibitors may of clinical use when administered in conjunction with thrombolysis to reduce or prevent such hemorrhagic complications.

#### Activation of MMP-9 by tPA

tPA has been shown to directly activate MMP-9 (Wang et al., 2003; Benarroch, 2007), with rt-PA treatment increasing MMP-9 activity in the serum of ischemic stroke patients (Golab et al., 2014). Such tPA-activation of MMP-9 may further amplify the MMP-9 response to stroke and involvement in injury pathways (Tsuji et al., 2005). Indeed, MMP-9 levels were shown to correlate with the levels of free radicals, measured as a marker of oxidative stress, this was observed in both tPA-treated and non-tPA treated patients (Kelly et al., 2006). However, tPA administration increases plasma MMP-9 levels, with co-administration of the free radical scavenger Edaravone having no effect on MMP-9 levels (Tsuruoka et al., 2014), although this may have been attributable to the low-dose Alteplase (0.6 mg/kg) used. An increased rate of HT on day 1 following acute ischemic stroke was observed in patients who underwent thrombolysis, compared to untreated controls (Carbone et al., 2015), with thromboylsis with tPA shown to increase venous blood levels of MMP-9 (Ning et al., 2006). Indeed, significantly higher levels of active MMP-9 have been observed in areas of HT, compared to both non-hemorrhagic, and non-ischemic tissue (Rosell et al., 2008). Indeed, MMP-9 levels were higher in patients that developed HT (Castellanos et al., 2003; Heo et al., 2003), with differences in MMP-9 levels even observed between symptomatic and asymptomatic HT (Castellanos et al., 2003). However, it has also been reported that neither baseline MMP-9 levels nor the rate of MMP-9 increase had any association with the risk of HT in the setting of ischemic stroke (Tsuruoka et al., 2014). Clearly this relationship requires further exploration. Nevertheless, it has been suggested that early HT is attributable to ROS, blood-derived MMP-9, and brain-derived MMP-2, whereas delayed HT is likely attributable to brain-derived MMP-9 and MMP-3, amongst other proteases, vascular remodeling and neuroinflammation (Jickling et al., 2014). Accordingly, early inhibition of MMP-9 may reduce hemorrhagic complications. MMP inhibitors may maintain the integrity of the BBB following stroke by limiting the access of tPA to brain parenchyma, thereby reducing hemorrhagic complications (Rosenberg and Yang, 2007).

### Post-mortem studies

Valuable insights into the changes in MMPs following human stroke was gained from post mortem studies of brain tissue from stroke patients. Fresh brain tissue from ischemic and hemorrhagic stroke patients examined within 6 h of death revealed higher levels of MMP-9, compared to control brains (Rosell et al., 2006). A further study by Rosell et al. (2008) of ischemic stroke patients with hemorrhagic complications revealed elevated MMP-9 levels within the stroke lesion. Within the infarct core, MMP-9 was localized to perivascular tissue and was associated with neutrophil infiltration whereas in the peri-infarct tissue microglial cells were found to highly express MMP-9 (Rosell et al., 2006). In hemorrhagic stroke, tissue surrounding the hematoma was shown to have increased levels of MMP-9. In both studies, no changes in MMP-2 levels were observed (Rosell et al., 2006, 2008). Neutrophils were identified as the main source of MMP-9 in areas of hemorrhage. Neutrophils were observed surrounding microvessels in conjunction with severe degradation of basal lamina type IV collagen and extravasation of blood into the surrounding brain parenchyma. As such, microvessel inflammation and MMP-9 appear to be key events associated with the development of hemorrhagic complications following ischemic stroke.

Overall, the clinical studies reveal that higher levels of MMP-9 are present in patients with acute ischemic stroke, compared with controls, and that this is a predictor for the development of hemorrhagic complications (both PH and HT), with MMP-9 levels correlating with larger infarct volume, increasing severity of stroke and poor functional outcome (Ramos-Fernandez et al., 2011). In this way, MMP-9 is proposed as a marker for ongoing brain ischemia and evolution of the stroke lesion, making it a potential candidate for inclusion in a stroke biomarker panel. As such, it is universally accepted that profound changes in MMP-9 expression and activity occur following ischemic stroke in both humans and animals. However, controversy still remains as to which cells are responsible for the bulk of the MMP-9 load following ischemic stroke.

## TIMP-1 following stroke

TIMPs are the endogenous inhibitors of MMPs present under normal conditions in tissues to regulate the activity of MMPs (Cunningham et al., 2005). However, just as MMP-9 levels are elevated following stroke, the expression of TIMP-1, the endogenous inhibitor of MMP-9, has also been shown to be dysregulated following stroke (Rivera et al., 2002). Furthermore, the MMP-9/TIMP-1 ratio has been proposed as a marker of stroke. Indeed, the correlation of MMP-9/TIMP-1 ratio with cerebral edema was even stronger than that of MMP-9 alone following ischemic stroke in rats (Li et al., 2013). Similar findings were observed in a clinical study with the relative increase in MMP-9/TIMP-1 ratio independently associated with symptomatic ICH in ischemic stroke patients (Piccardi et al., 2015). In keeping with the role of MMP-9 and TIMP-1 in the pathogenesis of stroke, mild hypothermia provided protection from ischemia/reperfusion injury via decreasing the expression of MMP-9 (Burk et al., 2008; Zhao et al., 2013) and TIMP-1 (Zhao et al., 2013).

Transient global cerebral ischemia led to TIMP-1 expression within 4 h of onset within the dentate gyrus of the hippocampus and progressed to involve other regions of the hippocampus including CA1 by 24 h post-stroke, a region especially vulnerable to ischemic injury. Such elevated TIMP-1 expression was observed in conjunction with elevated MMP-9 levels at 24 h following stroke onset (Fujimoto et al., 2008). Such increased expression of TIMP-1 was apparent in neurons and glial cells following global cerebral ischemia. This elevation in TIMP-1 may be a protective response to the ischemic insult to combat the increased levels of MMP-9 in an attempt to not only preserve endothelial barrier function but also participate in the long-term reorganization of the tissue following injury. Although the protective effects of TIMP-1 inhibition are thought to extend beyond simply its inhibition of MMP-9, as neuroprotection through TIMP-1 inhibition could not be reproduced with MMP inhibition alone (Chesler et al., 1995).

Rendering TIMP-1 inactive abolished the neuroprotective effects of TIMP-1 application to hippocampal cultures exposed to an excitotoxic insult (Tan et al., 2003). TIMP-1 knockout mice demonstrated significantly elevated Evan's Blue extravasation, indicative of profound BBB disruption and vasogenic edema, along with larger infarct volumes, in keeping with increased MMP-9 expression and activity in the absence of inhibition by TIMP-1 (Fujimoto et al., 2008). Presumably, most of the exogenously administered TIMPs act on endothelial cells and circulating immune cells, such as neutrophils, to inhibit MMP-9 activity, rather than the brain tissue itself as these molecules are unable to cross the intact BBB. Such a hypothesis certainly fits with the argument that neutrophils are the cellular source of MMP-9.

## Cellular source of MMP-9 following stroke

Increased MMP-9 levels have been well documented following both experimental and clinical stroke. However, the cellular source of this MMP-9, which is driving barrier permeability changes, cerebral edema, and hemorrhagic complications remains to be elucidated. A number of studies have sought to investigate what cells are releasing MMP-9 in stroke, with both peripheral and central candidates examined. A wide range of cell types have been shown to express MMP-9 following stroke, including neurons, microglia, and endothelial cells (Rosell et al., 2008). However, the main or initiating source of the MMP-9 remains unclear.

### Neutrophils and MMP-9

Following stroke, the chemokines and cytokines released in response to the ischemic insult promote the chemotaxis of inflammatory cells, such as neutrophils, to the ischemic site (Rodrigues and Granger, 2015). These cells transmigrate into the ischemic tissue where they contribute to tissue injury. In order to gain access to the brain tissue, the well-orchestrated expression of cellular adhesion molecules, integrins, and chemokines are required to enable neutrophils to roll along the endothelium, adhere to the endothelium and transmigrate through the endothelial cell barrier into brain tissue (Wang and Doerschuk, 2002; Schnoor and Parkos, 2008; Alcaide et al., 2009; Choi et al., 2009). Neutrophils are the main inflammatory cell type that responds to the inflammatory stimulus following stroke (Tang et al., 2006). Transmigration of neutrophils is thought to be reliant on three proteinases released from neutrophilic granules: neutrophil elastase (NE), collagenase, and gelatinase granules. These proteinases act in concert to degrade the ECM and allow passage of neutrophils into the tissue. Indeed, neutrophils are equipped with MMP-9 in secretory granules, which enables them to dissolving the ECM and basal lamina and “burrow” their way into the tissue (Choi et al., 2009). However, it has been suggested that neutrophils only require MMP-9 for stage 5 of transmigration only, where the neutrophils are migrating between the endothelial cells (Oda et al., 1995). Nonetheless, exocytosis of MMP-9 from gelatinase granules is likely to be important for the transmigration of neutrophils into tissues (Keck et al., 2002; Lee et al., 2003; Khandoga et al., 2006) with cell culture studies demonstrating that gelatinase activity is required for the migration of neutrophils through Matrigel and amnion membranes (Bakowski and Tschesche, 1992; Steadman et al., 1997). Once neutrophils gain access to the tissue they are then able to stimulate the release/production of MMP-9 from other cells types including resident brain cells, further perpetuating the effects of MMP-9 activity. Indeed, neutrophil neurotoxicity is dependent upon MMPs, reactive oxygen species (ROS) and cytokines such as tumor necrosis factor-α (TNF-α) (Nguyen et al., 2007).

The expression of MMP-9 by neutrophils occurs late in their development and thus following transcription, pro-MMP-9 is stored in cytoplasmic granules, along with neutrophil gelatinase-associated lipocalin, which protects the pro-MMP-9 molecules from degradation (Van den Steen et al., 2002). The presence of pre-made MMP-9 within these neutrophil granules allows the rapid release (within minutes) of MMP-9 into the extracellular milieu, as opposed to the mechanism of increased expression in other cell types that requires transcription and takes in excess of several hours (Opdenakker et al., 2001; Van den Steen et al., 2002). This suggests that neutrophils may be responsible for the early increases in MMP-9 observed in plasma following stroke. Following degranulation, pro-MMP-9 is released into the extracellular space where it is activated through removal of its pro-peptide by proteolysis (Opdenakker et al., 2001; Sternlicht and Werb, 2001). Although MMP-2 is generally constitutively expressed in most cell types, neutrophils are unique in that they do not express MMP-2, nor do they express TIMPs.

### Neutrophils as the cellular source of MMP-9 following stroke

Inflammatory cells have been implicated as important sources of MMP-9 in a variety of other pathological conditions including tumor progression, asthma, and hepatic injury (Yushchenko et al., 2000; Turba et al., 2007; Fujimura et al., 2009). Previous studies have suggested that neutrophils are the major source of MMP-9 acting on the BBB (Tang et al., 2006). Indeed, MMP-9 is expressed almost exclusively in neutrophils in peripheral blood (Tang et al., 2006) and increased BBB permeability induced by leukocyte-derived MMP-9 has been shown in ischemia-reperfusion injury to correlate with peak neutrophil infiltration (Sandoval and Witt, 2008), suggesting that neutrophils are a good candidate to be the main source of MMP-9 released following ischemic stroke.

#### Experimental studies on MMP-9 source

Investigation of the role of MMP-9 and neutrophils in cerebral ischemia has taken two approaches, either knockout of MMP-9 or depletion of neutrophils prior to induction of stroke. MMP-9 knockout significantly attenuates leukocyte recruitment into brain tissue following stroke, implicating a pro-inflammatory role for MMP-9 in the recruitment of leukocytes to reperfused brain tissue (Gidday et al., 2005). Gidday et al. (2005) was the first group to report on the cellular source of MMP-9 following stroke but since this paper there have been a number of other studies that have investigated whether neutrophils are the main contributors to the increased MMP-9 load observed following stroke. In their paper, Gidday et al. (2005) reported that depletion of neutrophils prior to induction of stroke markedly reduced vasogenic edema, collagen IV degradation and the extent of cerebral infarction, suggesting the MMP-9 promotes neutrophil transmigration into the brain and resultant injury. Such results provide compelling evidence for neutrophils as the source of MMP-9 following stroke.

One such study in support of the neutrophil-derived MMP-9 theory was carried out in rats with neutropenia treatment prior to induction of stroke to deplete neutrophils (Justicia et al., 2003). They reported an increase in 95 kDa MMP-9 in neutrophils and of 88 kDa MMP-9 in brain tissue following 1 h MCAO. Neutropenia treatment prior to stroke markedly reduced MMP-9 expression and prevented infiltration of neutrophils into the ischemia tissue. Treatment with an anti-intercellular adhesion molecule-1 (expressed on endothelial and immune cells) antibody, in addition to neutropenia, still produced low levels of MMP-9 expression, which was attributed to expression of MMP-9 by other cell types within the ischemic tissue. Accordingly, this group concluded that neutrophil infiltration was essential for the rise in MMP-9 expression and activity observed, and that neutrophils significantly contribute to the increase in MMP-9 in cerebral tissue through the release of pro-MMP-9.

In contrast, a few studies have reported that neutrophils are not the cellular source of MMP-9 following stroke (Maier et al., 2004; Harris et al., 2005; Zozulya et al., 2008). Specifically, 3 h MCAO was shown to produce a significant increase in MMP-9 and MMP-2 within the ischemic hemisphere, however prior neutrophil depletion did not affect MMP-9 protein levels, nor was there any benefit in terms of infarct volume, HT, cerebral edema, or functional outcome as measured by the neuroscore (Harris et al., 2005). Accordingly, this group proposed that neutrophils are not an important contributor to MMP-9 expression in the setting of cerebral ischemia and do not have a significant effect on neurovascular damage or neurological function and therefore is unlikely to be a key player in early microvascular damage and hemorrhagic complications following stroke. They suggest that neutrophils may be more important in ischemia models where there is a lower degree of injury, certainly the 3 h MCAO used in this study is at the severe end of the ischemia spectrum. Maier et al. (2004) found MMP-9 expression to be variable within the ischemic hemisphere and proposed that various cell types contribute differently in a region dependent manner. All myeloperoxidase (MPO, major component of neutrophil azurophilic granules)-positive cells were found to be MMP-9-postive and these cells were present in high numbers. Micgroglia were also shown to express MMP-9, but not all microglia were MMP-9 positive. MMP-9 immunoreactivity was clearly visible in vessels demonstrating Evan's blue extravasation. However, they did not observe any difference in the number of MPO positive cells over time, from the 24–72 h time-points, nor between the wild type of SOD2 animals examined. Furthermore, the MMP-9 response was shown to be bi-phasic, which did not coincide with neutrophil infiltration into the stroke lesion. Although not discounting the contribution of neutrophil-derived MMP-9 in barrier disruption and evolution of injury following stroke, this group suggest that neutrophils are not the primary source of MMP-9 following stroke and that other cell types such as microglia, astrocytes, and endothelial cells may be more appropriate targets for MMP inhibition. Such findings suggest that neutrophils may be the cells that initiate the release of MMP-9 in/directly from these other cell types, thereby potentiating MMP-9 release within ischemic tissue. As such, neutrophils may release MMP-9 locally at the BBB and do not need to migrate into the brain parenchyma to initiate the release of MMP-9 from resident brain cells to perpetuate the injury cascade. Indeed, studies of cultured endothelial cells showed that under normal conditions neutrophils weakly express MMP-9 but when co-cultured with pericytes an increase in MMP-9 secretion is observed, indicating that pericytes may be responsible for the increase in MMP-9 (Zozulya et al., 2008).

Neutrophils themselves are a significant source of ROS (Rodrigues and Granger, 2015). In a cell culture model, using neutrophil conditioned medium, neutrophils were shown to have high MMP-9 expression and release high levels of superoxide, hydrogen peroxide, and TNF-α (Nguyen et al., 2007). Treatment of the cells with the MMP-9 inhibitor GM6001 produced an 80% reduction in MMP-9 activity and an accompanying decrease in hydrogen peroxide and increase in TNF-α. Such findings indicate the neutrophil-derived MMP-9 can promote cell death by increasing the levels of ROS and also through the regulation of NE that increases endothelial cell death.

#### Clinical studies on MMP-9 source

Gene expression analysis of peripheral blood taken from ischemic stroke patients revealed that MMP-9 was significantly upregulated in neutrophils early after stroke (Tang et al., 2006). A human study measuring MMP-9 levels in the CSF and serum of controls and patients with various neurological disorders showed that MMP-9 was not expressed in the CSF of controls but was markedly increased in the CSF of patients. This increase in MMP-9 was attributable to an increased in neutrophils within the CSF (Yushchenko et al., 2000), whereas monocytes/macrophages and lymphocytes were only shown to be weak producers of MMP-9.

Not only have neutrophils been implicated as a source of MMP-9 following stroke which contributes to injury and barrier dysfunction, they have also been suggested to be the source of MMP-9 following tPA treatment. This was supported by the observation that tPA treatment can induce the release of pro-MMP-9 from ischemic brain (Justicia et al., 2003) and more recent observations that tPA can directly initiate the release of MMP-9 (Cuadrado et al., 2008), along with other MMPs and TIMPs. Given that tPA promotes neutrophil degranulation and the release of MMP-9. These inflammatory cells are good candidates to be the main source of MMP-9 post-stroke in the setting of tPA treatment, and may be responsible, at least in part, for tPA-induced hemorrhage (Cuadrado et al., 2008). In thrombolysed ischemic stroke patients a peak of neutrophil degranulation was observed 30 min following tPA administration (Carbone et al., 2015). The tPA-induced degranulation of neutrophils induced a combined release of the contents from primary (MPO and NE), secondary (collagenases such as MMP-8) and tertiary granules (MMP-9) (Carbone et al., 2015). Such increases in MMP-9 were seen in microvessels and neutrophils associated with the hemorrhagic tissue. Neutrophils were found to be an important source of MMP-9, as indicated by MPO staining, with total brain MMP-9 levels correlating with the number of MMP-9 positive neutrophils. In terms of HT, it appears that it is not the neutrophils adhered to the endothelium but rather those that transmigrate into the cerebral tissue that are responsible for such hemorrhagic complications (Gautier et al., 2009). As such, the location of the neutrophils, for example neutrophils within the cerebral vasculature, those localized to the BBB or neutrophils that have extravasated into the brain parenchyma, may determine the effect of MMP-9 release on the local tissue. However, others have suggested that neutrophils are not necessary for the acute development of HT (Harris et al., 2005). Human brain endothelial cells were shown to participate in MMP-mediated BBB breakdown during an ischemic insult, although their data did not support a role of MMP-9 in this process. Instead, MMP-2 was identified as the MMP that was elevated in ischemia.

## Comparison between experimental and clinical data

The experimental and clinical literature is in agreeance that profound elevations in MMP-9 occur early following stroke that can hasten and exacerbate injury. Specifically, in the experimental studies, elevations in MMP-9 are observed as early as 3 h following stroke (Shigemori et al., 2006) and persist for days following stroke onset. Such alterations in MMP-9 are associated with increased BBB permeability (Romanic et al., 1998; Rosenberg et al., 1998; Asahi et al., 2001b; Sandoval and Witt, 2008), the development of cerebral edema (Li et al., 2013), hemorrhagic transformation (Romanic et al., 1998; Rosenberg et al., 1998; Asahi et al., 2001b; Sandoval and Witt, 2008), increased infarct volume (Romanic et al., 1998; Asahi et al., 2000, 2001b; Lee et al., 2003; Hu et al., 2009; Wang et al., 2009; Li et al., 2013), and poor outcomes (Romanic et al., 1998; Asahi et al., 2000, 2001b; Lee et al., 2003; Hu et al., 2009; Wang et al., 2009; Li et al., 2013) in animal models. Indeed, the clinical studies report a comparable picture with MMP-9 levels elevated as early as 6 h post-stroke in post-mortem tissue (Rosell et al., 2006) and within 12 h in patients (Reynolds et al., 2003) with such alterations and persisting for many days thereafter with these changes associated with large infarct volumes (Horstmann et al., 2003; Rosell et al., 2005; Sotgiu et al., 2006; Vukasovic et al., 2006), hemorrhagic transformation of the infarct (both with and without tPA treatment), and poor outcomes (Rosell et al., 2005; Ning et al., 2006; Rodríguez-Yáñez et al., 2006; Vukasovic et al., 2006) in clinical stroke patients. Furthermore, the experimental and clinical literature also largely agree that neutrophils are the source of MMP-9 (Yushchenko et al., 2000; Justicia et al., 2003; Gidday et al., 2005; Cuadrado et al., 2008; Gautier et al., 2009) which is contributing to hastening of the ischemic injury including infarct extension and the development of complications such as hemorrhagic transformation.

## Conclusions

**Figure 1:**
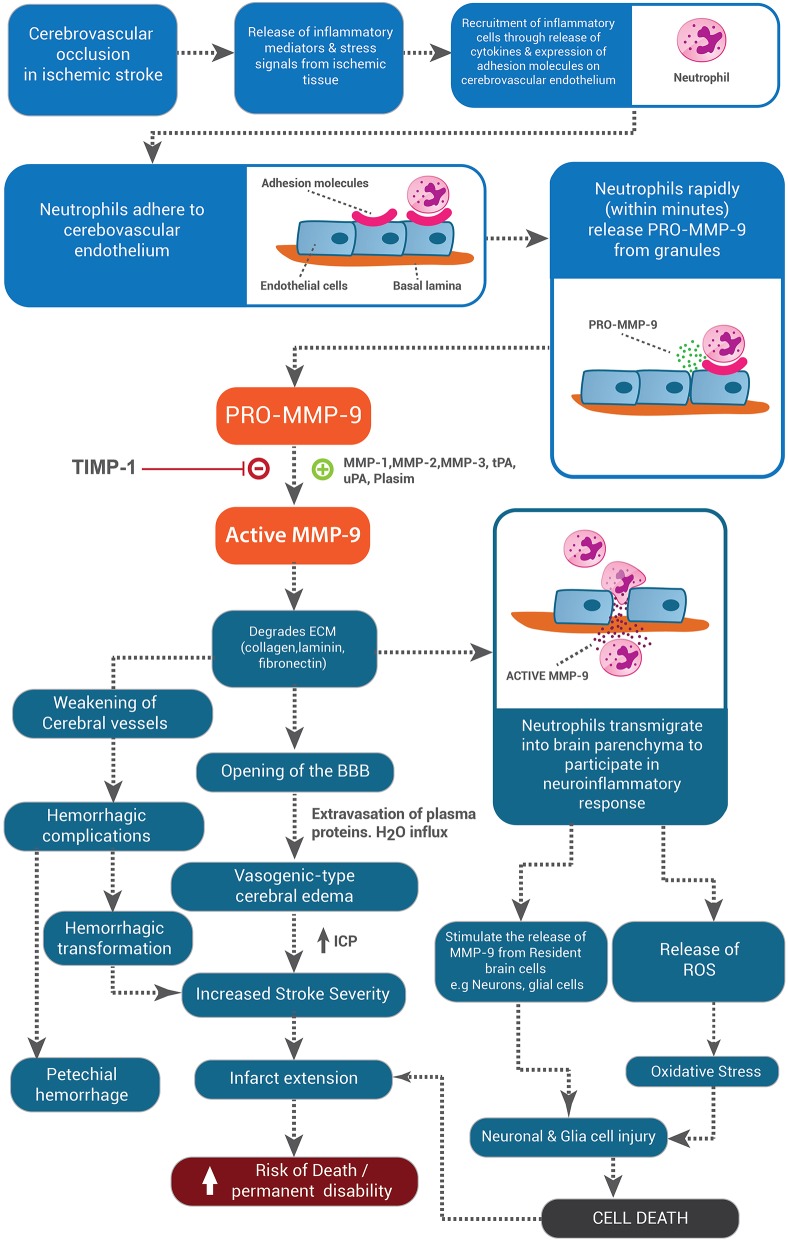
**The role of MMP-9 in exacerbating injury pathways in ischemic stroke**. MMP-9 released from neutrophils allows these cells to transmigrate into the brain tissue, where they release MMP-9 and other deleterious agents such as ROS, thereby stimulating the release of MMP-9 from resident brain cells and contributing to the cell injury and cell death pathways. Neutrophil-derived MMP-9 actively degrades components of the BBB leading to the development of cerebral edema and hemorrhagic transformation, both of which worsen stroke severity, lead to infarct extension and increase the risk of death and disability post-stroke.

We propose a likely model to explain the sequential breakdown and repair of the BBB following stroke (Figure 1). Early after stroke (12–48 h) neutrophils adhere to the cerebrovascular endothelium where they release MMP-9 that degrades the basal lamina of the endothelial cells and astrocytes. This enables migration of neutrophils into the ischemic tissue and subsequent opening of the BBB results in the formation of cerebral edema. At the ischemic core, MMP-9 may be released by both infiltrating neutrophils and resident microglia. Neutrophil-derived MMP-9 degrades the BBB, whilst the microglia-derived MMP-9 acts in concert with other molecules to produce neuronal and glial cell death and microglia likely undergo apoptosis in this environment. Upon restoration of blood flow, repair of the BBB may be initiated at the margins of the infarction. At this stage, all cells, including endothelial cells, glia, and neurons, may release MMPs and other proteases at low levels in order to remodel the basement membrane and restore cell-cell contacts to allow for angiogenesis and gliogenesis to occur in order to re-establish the BBB. Therefore, manipulation of the MMP-TIMP system following stroke needs to be well orchestrated to prevent tissue injury early but also assist in tissue remodeling late after the event and the therapeutic window available to limit BBB destruction following stroke is quite short, after which reparative functions would need to predominate.

In conclusion, a number of experimental studies suggest that neutrophils are in fact the main cellular course of MMP-9 following stroke (not resident brain cells) and that these cells use MMP-9 to transmigrate into ischemic tissue, contribute to ischemic injury in the form of ECM degradation, BBB permeability changes, vasogenic edema, and HT, whilst also stimulating the release of MMP-9 from resident brain cells such as neurons and microglia. Clearly, the interaction of action of leukocytes at the BBB following stroke requires further investigation to elucidate the contribution of neutrophil-derived to ECM degradation, BBB disruption and subsequent vasogenic edema, hemorrhage and parenchymal injury.

Many studies have focused on the protection of neurons as a therapeutic target. However, given that such a small percentage of all cells in the brain are neurons it seems more appropriate to consider protection across many cell types. Accordingly, investigation of the neurovascular unit and the complex interplay of endothelial cells, pericytes, astrocytes, and infiltrating leukocytes is likely to yield more appropriate therapeutic targets. Information regarding the relationship between MMP-9 and neutrophils may facilitate understanding of the mechanisms involved in BBB breakdown following stroke and ultimately guide therapeutic intervention.

## Author contributions

All authors listed, have made substantial, direct and intellectual contribution to the work, and approved it for publication.

### Conflict of interest statement

The authors declare that the research was conducted in the absence of any commercial or financial relationships that could be construed as a potential conflict of interest.

## References

[B1] Adibhatla R. M. Hatcher J. F. (2008). Tissue plasminogen activator (tPA) and matrix metalloproteinases in the pathogenesis of stroke: therapeutic strategies. CNS Neurol. Disord. Drug Targets 7, 243–253. 10.2174/187152708784936608 18673209 PMC2562687

[B2] Agrawal S. M. Lau L. Yong V. W. (2008). MMPs in the central nervous system: where the good guys go bad. Semin. Cell Dev. Biol. 19, 42–51. 10.1016/j.semcdb.2007.06.003 17646116

[B3] Alcaide P. Auerbach S. Luscinskas F. W. (2009). Neutrophil recruitment under shear flow: it's all about endothelial cell rings and gaps. Microcirculation 16, 43–57. 10.1080/10739680802273892 18720226 PMC2726622

[B4] Amantea D. Certo M. Russo R. Bagetta G. Corasaniti M. T. Tassorelli C. (2014). Early reperfusion injury is associated to MMP2 and IL-1beta elevation in cortical neurons of rats subjected to middle cerebral artery occlusion. Neuroscience 277, 755–763. 10.1016/j.neuroscience.2014.07.064 25108165

[B5] Amantea D. Corasaniti M. T. Mercuri N. B. Bernardi G. Bagetta G. (2008). Brain regional and cellular localization of gelatinase activity in rat that have undergone transient middle cerebral artery occlusion. Neuroscience 152, 8–17. 10.1016/j.neuroscience.2007.12.030 18255236

[B6] Anthony D. C. Ferguson B. Matyzak M. K. Miller K. M. Esiri M. M. Perry V. H. (1997). Differential matrix metalloproteinase expression in cases of multiple sclerosis and stroke. Neuropathol. Appl. Neurobiol. 23, 406–415. 10.1111/j.1365-2990.1997.tb01315.x 9364466

[B7] Asahi M. Asahi K. Wang X. Lo E. H. (2000). Reduction of tissue plasminogen activator-induced hemorrhage and brain injury by free radical spin trapping after embolic focal cerebral ischemia in rats. J. Cereb. Blood Flow Metab. 20, 452–457. 10.1097/00004647-200003000-00002 10724108

[B8] Asahi M. Sumii T. Fini M. E. Itohara S. Lo E. H. (2001a). Matrix metalloproteinase 2 gene knockout has no effect on acute brain injury after focal ischemia. Neuroreport 12, 3003–3007. 10.1097/00001756-200109170-00050 11588620

[B9] Asahi M. Wang X. Mori T. Sumii T. Jung J. C. Moskowitz M. A.. (2001b). Effects of matrix metalloproteinase-9 gene knock-out on the proteolysis of blood-brain barrier and white matter components after cerebral ischemia. J. Neurosci. 21, 7724–7732. 11567062 10.1523/JNEUROSCI.21-19-07724.2001 PMC6762894

[B10] Bajor M. Kaczmarek L. (2013). Proteolytic remodeling of the synaptic cell adhesion molecules (CAMs) by metzincins in synaptic plasticity. Neurochem. Res. 38, 1113–1121. 10.1007/s11064-012-0919-6 23124395 PMC3653053

[B11] Bakowski B. Tschesche H. (1992). Migration of polymorphonuclear leukocytes through human amnion membrane–a scanning electron microscopic study. Biol. Chem. Hoppe Seyler 373, 529–546. 1515084

[B12] Bauer A. T. Bürgers H. F. Rabie T. Marti H. H. (2010). Matrix metalloproteinase-9 mediates hypoxia-induced vascular leakage in the brain via tight junction rearrangement. J. Cereb. Blood Flow Metab. 30, 837–848. 10.1038/jcbfm.2009.248 19997118 PMC2949161

[B13] Benarroch E. E. (2007). Tissue plasminogen activator: beyond thrombolysis. Neurology 69, 799–802. 10.1212/01.wnl.0000269668.08747.78 17709713

[B14] Burk J. Burggraf D. Vosko M. Dichgans M. Hamann G. F. (2008). Protection of cerebral microvasculature after moderate hypothermia following experimental focal cerebral ischemia in mice. Brain Res. 1226, 248–255. 10.1016/j.brainres.2008.06.015 18586014

[B15] Candelario-Jalil E. Yang Y. Rosenberg G. A. (2009). Diverse roles of matrix metalloproteinases and tissue inhibitors of metalloproteinases in neuroinflammation and cerebral ischemia. Neuroscience 158, 983–994. 10.1016/j.neuroscience.2008.06.025 18621108 PMC3584171

[B16] Carbone F. Vuilleumier N. Bertolotto M. Burger F. Galan K. Roversi G.. (2015). Treatment with recombinant tissue plasminogen activator (r-TPA) induces neutrophil degranulation *in vitro* via defined pathways. Vascul. Pharmacol. 64, 16–27. 10.1016/j.vph.2014.11.007 25530154

[B17] Castellanos M. Leira R. Serena J. Pumar J. M. Lizasoain I. Castillo J.. (2003). Plasma metalloproteinase-9 concentration predicts hemorrhagic transformation in acute ischemic stroke. Stroke 34, 40–46. 10.1161/01.STR.0000046764.57344.31 12511748

[B18] Castellanos M. Sobrino T. Millán M. García M. Arenillas J. Nombela F.. (2007). Serum cellular fibronectin and matrix metalloproteinase-9 as screening biomarkers for the prediction of parenchymal hematoma after thrombolytic therapy in acute ischemic stroke: a multicenter confirmatory study. Stroke 38, 1855–1859. 10.1161/STROKEAHA.106.481556 17478737

[B19] Chang D. I. Hosomi N. Lucero J. Heo J. H. Abumiya T. Mazar A. P.. (2003). Activation systems for latent matrix metalloproteinase-2 are upregulated immediately after focal cerebral ischemia. J. Cereb. Blood Flow Metab. 23, 1408–1419. 10.1097/01.WCB.0000091765.61714.30 14663336

[B20] Chesler L. Golde D. W. Bersch N. Johnson M. D. (1995). Metalloproteinase inhibition and erythroid potentiation are independent activities of tissue inhibitor of metalloproteinases-1. Blood 86, 4506–4515. 8541540

[B21] Choi E. Y. Santoso S. Chavakis T. (2009). Mechanisms of neutrophil transendothelial migration. Front. Biosci. 14, 1596–1605. 10.2741/3327 19273149 PMC2672407

[B22] Clark A. W. Krekoski C. A. Bou S. S. Chapman K. R. Edwards D. R. (1997). Increased gelatinase A (MMP-2) and gelatinase B (MMP-9) activities in human brain after focal ischemia. Neurosci. Lett. 238, 53–56. 10.1016/S0304-3940(97)00859-8 9464653

[B23] Clark I. M. Swingler T. E. Sampieri C. L. Edwards D. R. (2008). The regulation of matrix metalloproteinases and their inhibitors. Int. J. Biochem. Cell Biol. 40, 1362–1378. 10.1016/j.biocel.2007.12.006 18258475

[B24] Conant K. Allen M. Lim S. T. (2015). Activity dependent CAM cleavage and neurotransmission. Front. Cell. Neurosci. 9:305. 10.3389/fncel.2015.00305 26321910 PMC4531370

[B25] Copin J. C. Goodyear M. C. Gidday J. M. Shah A. R. Gascon E. Dayer A.. (2005). Role of matrix metalloproteinases in apoptosis after transient focal cerebral ischemia in rats and mice. Eur. J. Neurosci. 22, 1597–1608. 10.1111/j.1460-9568.2005.04367.x 16197500

[B26] Cuadrado E. Ortega L. Hernández-Guillamon M. Penalba A. Fernández-Cadenas I. Rosell A.. (2008). Tissue plasminogen activator (t-PA) promotes neutrophil degranulation and MMP-9 release. J. Leukoc. Biol. 84, 207–214. 10.1189/jlb.0907606 18390930

[B27] Cuadrado E. Rosell A. Borrell-Pagès M. García-Bonilla L. Hernández-Guillamon M. Ortega-Aznar A.. (2009). Matrix metalloproteinase-13 is activated and is found in the nucleus of neural cells after cerebral ischemia. J. Cereb. Blood Flow Metab. 29, 398–410. 10.1038/jcbfm.2008.130 18985055

[B28] Cunningham L. A. Wetzel M. Rosenberg G. A. (2005). Multiple roles for MMPs and TIMPs in cerebral ischemia. Glia 50, 329–339. 10.1002/glia.20169 15846802

[B29] Dejonckheere E. Vandenbroucke R. E. Libert C. (2011). Matrix metalloproteinases as drug targets in ischemia/reperfusion injury. Drug Discov. Today 16, 762–778. 10.1016/j.drudis.2011.06.009 21745586

[B30] Fanjul-Fernández M. Folgueras A. R. Cabrera S. López-Otín C. (2010). Matrix metalloproteinases: evolution, gene regulation and functional analysis in mouse models. Biochim. Biophys. Acta 1803, 3–19. 10.1016/j.bbamcr.2009.07.004 19631700

[B31] Fernandez-Patron C. Zouki C. Whittal R. Chan J. S. Davidge S. T. Filep J. G. (2001). Matrix metalloproteinases regulate neutrophil-endothelial cell adhesion through generation of endothelin-1[1-32]. FASEB J. 15, 2230–2240. 10.1096/fj.01-0178com 11641250

[B32] Ferry G. Lonchampt M. Pennel L. de Nanteuil G. Canet E. Tucker G. C. (1997). Activation of MMP-9 by neutrophil elastase in an *in vivo* model of acute lung injury. FEBS Lett. 402, 111–115. 10.1016/S0014-5793(96)01508-6 9037177

[B33] Fiorelli M. Bastianello S. von Kummer R. del Zoppo G. J. Larrue V. Lesaffre E.. (1999). Hemorrhagic transformation within 36 hours of a cerebral infarct: relationships with early clinical deterioration and 3-month outcome in the European Cooperative Acute Stroke Study I (ECASS I) cohort. Strok 30, 2280–2284. 10.1161/01.STR.30.11.2280 10548658

[B34] Fujimoto M. Takagi Y. Aoki T. Hayase M. Marumo T. Gomi M.. (2008). Tissue inhibitor of metalloproteinases protect blood-brain barrier disruption in focal cerebral ischemia. J. Cereb. Blood Flow Metab. 28, 1674–1685. 10.1038/jcbfm.2008.59 18560439

[B35] Fujimura M. Gasche Y. Morita-Fujimura Y. Massengale J. Kawase M. Chan P. H. (1999). Early appearance of activated matrix metalloproteinase-9 and blood-brain barrier disruption in mice after focal cerebral ischemia and reperfusion. Brain Res. 842, 92–100. 10.1016/S0006-8993(99)01843-0 10526099

[B36] Fujimura M. Watanabe M. Narisawa A. Shimizu H. Tominaga T. (2009). Increased expression of serum Matrix Metalloproteinase-9 in patients with moyamoya disease. Surg. Neurol. 72, 476–480. 10.1016/j.surneu.2008.10.009 19147196

[B37] Gasche Y. Copin J. C. Sugawara T. Fujimura M. Chan P. H. (2001). Matrix metalloproteinase inhibition prevents oxidative stress-associated blood-brain barrier disruption after transient focal cerebral ischemia. J. Cereb. Blood Flow Metab. 21, 1393–1400. 10.1097/00004647-200112000-00003 11740200

[B38] Gasche Y. Fujimura M. Morita-Fujimura Y. Copin J. C. Kawase M. Massengale J.. (1999). Early appearance of activated matrix metalloproteinase-9 after focal cerebral ischemia in mice: a possible role in blood-brain barrier dysfunction. J. Cereb. Blood Flow Metab. 19, 1020–1028. 10.1097/00004647-199909000-00010 10478654

[B39] Gasche Y. Soccal P. M. Kanemitsu M. Copin J. C. (2006). Matrix metalloproteinases and diseases of the central nervous system with a special emphasis on ischemic brain. Front. Biosci. 11, 1289–1301. 10.2741/1883 16368516

[B40] Gautier S. Ouk T. Petrault O. Caron J. Bordet R. (2009). Neutrophils contribute to intracerebral haemorrhages after treatment with recombinant tissue plasminogen activator following cerebral ischaemia. Br. J. Pharmacol. 156, 673–679. 10.1111/j.1476-5381.2009.00068.x 19210512 PMC2697703

[B41] Gidday J. M. Gasche Y. G. Copin J. C. Shah A. R. Perez R. S. Shapiro S. D.. (2005). Leukocyte-derived matrix metalloproteinase-9 mediates blood-brain barrier breakdown and is proinflammatory after transient focal cerebral ischemia. Am. J. Physiol. Heart Circ. Physiol. 289, H558–H568. 10.1152/ajpheart.01275.2004 15764676

[B42] Golab P. Boguszewska-Czubara A. Kielbus M. Kurzepa J. (2014). The rtPA increases MMP-9 activity in serum during ischaemic stroke. Neurol. Neurochir. Pol. 48, 309–314. 10.1016/j.pjnns.2014.07.012 25440008

[B43] Gu Z. Kaul M. Yan B. Kridel S. J. Cui J. Strongin A.. (2002). S-nitrosylation of matrix metalloproteinases: signaling pathway to neuronal cell death. Science 297, 1186–1190. 10.1126/science.1073634 12183632

[B44] Gurney K. J. Estrada E. Y. Rosenberg G. A. (2006). Blood-brain barrier disruption by stromelysin-1 facilitates neutrophil infiltration in neuroinflammation. Neurobiol. Dis. 23, 87–96. 10.1016/j.nbd.2006.02.006 16624562

[B45] Gürsoy-Ozdemir Y. Bolay H. Saribas O. Dalkara T. (2000). Role of endothelial nitric oxide generation and peroxynitrite formation in reperfusion injury after focal cerebral ischemia. Stroke 31, 1974–1980. 10.1161/01.STR.31.8.1974 10926966

[B46] Hacke W. Schwab S. Horn M. Spranger M. De Georgia M. von Kummer R. (1996). ‘Malignant’ middle cerebral artery territory infarction: clinical course and prognostic signs. Arch. Neurol. 53, 309–315. 10.1001/archneur.1996.00550040037012 8929152

[B47] Harris A. K. Ergul A. Kozak A. Machado L. S. Johnson M. H. Fagan S. C. (2005). Effect of neutrophil depletion on gelatinase expression, edema formation and hemorrhagic transformation after focal ischemic stroke. BMC Neurosci. 6:49. 10.1186/1471-2202-6-49 16078993 PMC1190186

[B48] Heo J. H. Kim S. H. Lee K. Y. Kim E. H. Chu C. K. Nam J. M. (2003). Increase in plasma matrix metalloproteinase-9 in acute stroke patients with thrombolysis failure. Stroken 34, e48–e50. 10.1161/01.str.0000073788.81170.1c 12750540

[B49] Heo J. H. Lucero J. Abumiya T. Koziol J. A. Copeland B. R. del Zoppo G. J. (1999). Matrix metalloproteinases increase very early during experimental focal cerebral ischemia. J. Cereb. Blood Flow Metab. 19, 624–633. 10.1097/00004647-199906000-00005 10366192

[B50] Horstmann S. Kalb P. Koziol J. Gardner H. Wagner S. (2003). Profiles of matrix metalloproteinases, their inhibitors, and laminin in stroke patients: influence of different therapies. Stroken 34, 2165–2170. 10.1161/01.STR.0000088062.86084.F2 12907822

[B51] Hu Q. Chen C. Yan J. Yang X. Shi X. Zhao J.. (2009). Therapeutic application of gene silencing MMP-9 in a middle cerebral artery occlusion-induced focal ischemia rat model. Exp. Neurol. 216, 35–46. 10.1016/j.expneurol.2008.11.007 19073180

[B52] Inzitari D. Giusti B. Nencini P. Gori A. M. Nesi M. Palumbo V.. (2013). MMP9 variation after thrombolysis is associated with hemorrhagic transformation of lesion and death. Stroke 44, 2901–2903. 10.1161/STROKEAHA.113.002274 23908067

[B53] Jickling G. C. Liu D. Stamova B. Ander B. P. Zhan X. Lu A.. (2014). Hemorrhagic transformation after ischemic stroke in animals and humans. J. Cereb. Blood Flow Metab. 34, 185–199. 10.1038/jcbfm.2013.203 24281743 PMC3915212

[B54] Justicia C. Panés J. Solé S. Cervera A. Deulofeu R. Chamorro A.. (2003). Neutrophil infiltration increases matrix metalloproteinase-9 in the ischemic brain after occlusion/reperfusion of the middle cerebral artery in rats. J. Cereb. Blood Flow Metab. 23, 1430–1440. 10.1097/01.WCB.0000090680.07515.C8 14663338

[B55] Keck T. Balcom J. H. IV Fernández-del Castillo C. Antoniu B. A. Warshaw A. L. (2002). Matrix metalloproteinase-9 promotes neutrophil migration and alveolar capillary leakage in pancreatitis-associated lung injury in the rat. Gastroenterology 122, 188–201. 10.1053/gast.2002.30348 11781293

[B56] Kelly M. A. Shuaib A. Todd K. G. (2006). Matrix metalloproteinase activation and blood-brain barrier breakdown following thrombolysis. Exp. Neurol. 200, 38–49. 10.1016/j.expneurol.2006.01.032 16624294

[B57] Khandoga A. Kessler J. S. Hanschen M. Khandoga A. G. Burggraf D. Reichel C.. (2006). Matrix metalloproteinase-9 promotes neutrophil and T cell recruitment and migration in the postischemic liver. J. Leukoc. Biol. 79, 1295–1305. 10.1189/jlb.0805468 16551680

[B58] Kim Y. S. Kim S. S. Cho J. J. Choi D. H. Hwang O. Shin D. H.. (2005). Matrix metalloproteinase-3: a novel signaling proteinase from apoptotic neuronal cells that activates microglia. J. Neurosci. 25, 3701–3711. 10.1523/JNEUROSCI.4346-04.2005 15814801 PMC6725382

[B59] Kuroiwa T. Cahn R. Juhler M. Goping G. Campbell G. Klatzo I. (1985). Role of extracellular proteins in the dynamics of vasogenic brain edema. Acta Neuropathol. 66, 3–11. 10.1007/BF00698288 3993334

[B60] Kurzepa J. Szczepanska-Szerej A. Stryjecka-Zimmer M. Malecka-Massalska T. Stelmasiak Z. (2006). Simvastatin could prevent increase of the serum MMP-9/TIMP-1 ratio in acute ischaemic stroke. Folia Biol. 52, 181–183. 17184595 10.14712/fb2006052060181

[B61] Lapchak P. A. Chapman D. F. Zivin J. A. (2000). Metalloproteinase inhibition reduces thrombolytic (tissue plasminogen activator)-induced hemorrhage after thromboembolic stroke. Stroke 31, 3034–3040. 10.1161/01.STR.31.12.3034 11108768

[B62] Lee K. S. Jin S. M. Kim H. J. Lee Y. C. (2003). Matrix metalloproteinase inhibitor regulates inflammatory cell migration by reducing ICAM-1 and VCAM-1 expression in a murine model of toluene diisocyanate-induced asthma. J. Allergy Clin. Immunol. 111, 1278–1284. 10.1067/mai.2003.1501 12789230

[B63] Lenglet S. Montecucco F. Mach F. (2015). Role of matrix metalloproteinases in animal models of ischemic stroke. Curr. Vasc. Pharmacol. 13, 161–166. 10.2174/15701611113116660161 24188490

[B64] Lenglet S. Montecucco F. Mach F. Schaller K. Gasche Y. Copin J. C. (2014). Analysis of the expression of nine secreted matrix metalloproteinases and their endogenous inhibitors in the brain of mice subjected to ischaemic stroke. Thromb. Haemost. 112, 363–378. 10.1160/TH14-01-0007 24671655

[B65] Li D. D. Song J. N. Huang H. Guo X. Y. An J. Y. Zhang M.. (2013). The roles of MMP-9/TIMP-1 in cerebral edema following experimental acute cerebral infarction in rats. Neurosci. Lett. 550, 168–172. 10.1016/j.neulet.2013.06.034 23819982

[B66] Lucivero V. Prontera M. Mezzapesa D. M. Petruzzellis M. Sancilio M. Tinelli A.. (2007). Different roles of matrix metalloproteinases-2 and -9 after human ischaemic stroke. Neurol. Sci. 28, 165–170. 10.1007/s10072-007-0814-0 17690845

[B67] Lukes A. Mun-Bryce S. Lukes M. Rosenberg G. A. (1999). Extracellular matrix degradation by metalloproteinases and central nervous system diseases. Mol. Neurobiol. 19, 267–284. 10.1007/BF02821717 10495107

[B68] Lynch J. R. Blessing R. White W. D. Grocott H. P. Newman M. F. Laskowitz D. T. (2004). Novel diagnostic test for acute stroke. Stroke 35, 57–63. 10.1161/01.STR.0000105927.62344.4C 14671250

[B69] Machado L. S. Sazonova I. Y. Kozak A. Wiley D. C. El-Remessy A. B. Ergul A. (2009). Minocycline and tissue-type plasminogen activator for stroke: assessment of interaction potential. Stroke 40, 3028–3033. 10.1161/STROKEAHA.109.556852 19628804 PMC2754038

[B70] Maier C. M. Hsieh L. Yu F. Bracci P. Chan P. H. (2004). Matrix metalloproteinase-9 and myeloperoxidase expression: quantitative analysis by antigen immunohistochemistry in a model of transient focal cerebral ischemia. Stroke 35, 1169–1174. 10.1161/01.STR.0000125861.55804.f2 15060315

[B71] Manabe S. Gu Z. Lipton S. A. (2005). Activation of matrix metalloproteinase-9 via neuronal nitric oxide synthase contributes to NMDA-induced retinal ganglion cell death. Invest. Ophthalmol. Vis. Sci. 46, 4747–4753. 10.1167/iovs.05-0128 16303975

[B72] McCarthy S. M. Bove P. F. Matthews D. E. Akaike T. van der Vliet A. (2008). Nitric oxide regulation of MMP-9 activation and its relationship to modifications of the cysteine switch. Biochemistry 47, 5832–5840. 10.1021/bi702496v 18452312 PMC3030771

[B73] Moldes O. Sobrino T. Millán M. Castellanos M. Pérez de la Ossa N. Leira R.. (2008). High serum levels of endothelin-1 predict severe cerebral edema in patients with acute ischemic stroke treated with t-PA. Stroke 39, 2006–2010. 10.1161/STROKEAHA.107.495044 18436890

[B74] Montaner J. Alvarez-Sabín J. Barberá G. Anglés A. Molina C. Abilleira S.. (2001a). [Correlation between the expression of proinflammatory cytokines and matrix metalloproteinases in the acute phase of an ischemic stroke]. Rev. Neurol. 33, 115–118. 11562868

[B75] Montaner J. Alvarez-Sabín J. Molina C. Anglés A. Abilleira S. Arenillas J.. (2001b). Matrix metalloproteinase expression after human cardioembolic stroke: temporal profile and relation to neurological impairment. Stroke 32, 1759–1766. 10.1161/01.STR.32.8.1759 11486102

[B76] Montaner J. Alvarez-Sabín J. Molina C. A. Anglés A. Abilleira S. Arenillas J.. (2001c). Matrix metalloproteinase expression is related to hemorrhagic transformation after cardioembolic stroke. Stroke 32, 2762–2767. 10.1161/hs1201.99512 11739970

[B77] Montaner J. Fernández-Cadenas I. Molina C. A. Monasterio J. Arenillas J. F. Ribo M.. (2003a). Safety profile of tissue plasminogen activator treatment among stroke patients carrying a common polymorphism (C-1562T) in the promoter region of the matrix metalloproteinase-9 gene. Stroke 34, 2851–2855. 10.1161/01.STR.0000098648.54429.1C 14605329

[B78] Montaner J. Molina C. A. Monasterio J. Abilleira S. Arenillas J. F. Ribó M.. (2003b). Matrix metalloproteinase-9 pretreatment level predicts intracranial hemorrhagic complications after thrombolysis in human stroke. Circulation 107, 598–603. 10.1161/01.CIR.0000046451.38849.90 12566373

[B79] Montaner J. Perea-Gainza M. Delgado P. Ribó M. Chacón P. Rosell A.. (2008). Etiologic diagnosis of ischemic stroke subtypes with plasma biomarkers. Stroken 39, 2280–2287. 10.1161/STROKEAHA.107.505354 18535284

[B80] Mun-Bryce S. Rosenberg G. A. (1998). Matrix metalloproteinases in cerebrovascular disease. J. Cereb. Blood Flow Metab. 18, 1163–1172. 10.1097/00004647-199811000-00001 9809504

[B81] Murata Y. Rosell A. Scannevin R. H. Rhodes K. J. Wang X. Lo E. H. (2008). Extension of the thrombolytic time window with minocycline in experimental stroke. Stroke 39, 3372–3377. 10.1161/STROKEAHA.108.514026 18927459 PMC3705574

[B82] Nguyen H. X. O'Barr T. J. Anderson A. J. (2007). Polymorphonuclear leukocytes promote neurotoxicity through release of matrix metalloproteinases, reactive oxygen species, and TNF-alpha. J. Neurochem. 102, 900–912. 10.1111/j.1471-4159.2007.04643.x 17561941

[B83] Ning M. Furie K. L. Koroshetz W. J. Lee H. Barron M. Lederer M.. (2006). Association between tPA therapy and raised early matrix metalloproteinase-9 in acute stroke. Neurology 66, 1550–1555. 10.1212/01.wnl.0000216133.98416.b4 16717217

[B84] Oda T. Katori M. Hatanaka K. Nagai Y. (1995). Inhibition of neutrophil migration by a selective inhibitor of matrix metalloproteinase: analysis by intravital microscopy. Mediators Inflamm. 4, 133–137. 10.1155/S0962935195000238 18475630 PMC2365620

[B85] Opdenakker G. Van den Steen P. E. Dubois B. Nelissen I. Van Coillie E. Masure S.. (2001). Gelatinase B functions as regulator and effector in leukocyte biology. J. Leukoc. Biol. 69, 851–859. 11404367

[B86] Orbe J. Barrenetxe J. Rodriguez J. A. Vivien D. Orset C. Parks W. C.. (2011). Matrix metalloproteinase-10 effectively reduces infarct size in experimental stroke by enhancing fibrinolysis via a thrombin-activatable fibrinolysis inhibitor-mediated mechanism. Circulation 124, 2909–2919. 10.1161/CIRCULATIONAHA.111.047100 22104553

[B87] Piccardi B. Palumbo V. Nesi M. Nencini P. Gori A. M. Giusti B.. (2015). Unbalanced metalloproteinase-9 and tissue inhibitors of metalloproteinases ratios predict hemorrhagic transformation of lesion in ischemic stroke patients treated with thrombolysis: results from the MAGIC study. Front. Neurol. 6:121. 10.3389/fneur.2015.00121 26074872 PMC4445323

[B88] Planas A. M. Solé S. Justicia C. (2001). Expression and activation of matrix metalloproteinase-2 and -9 in rat brain after transient focal cerebral ischemia. Neurobiol. Dis. 8, 834–846. 10.1006/nbdi.2001.0435 11592852

[B89] Ra H. J. Parks W. C. (2007). Control of matrix metalloproteinase catalytic activity. Matrix Biol. 26, 587–596. 10.1016/j.matbio.2007.07.001 17669641 PMC2246078

[B90] Ramos-Fernandez M. Bellolio M. F. Stead L. G. (2011). Matrix metalloproteinase-9 as a marker for acute ischemic stroke: a systematic review. J. Stroke Cerebrovasc. Dis. 20, 47–54. 10.1016/j.jstrokecerebrovasdis.2009.10.008 21044610

[B91] Ranasinghe H. S. Scheepens A. Sirimanne E. Mitchell M. D. Williams C. E. Fraser M. (2012). Inhibition of MMP-9 activity following hypoxic ischemia in the developing brain using a highly specific inhibitor. Dev. Neurosci. 34, 417–427. 10.1159/000343257 23171520

[B92] Reynolds M. A. Kirchick H. J. Dahlen J. R. Anderberg J. M. McPherson P. H. Nakamura K. K.. (2003). Early biomarkers of stroke. Clin. Chem. 49, 1733–1739. 10.1373/49.10.1733 14500614

[B93] Rivera S. Ogier C. Jourquin J. Timsit S. Szklarczyk A. W. Miller K.. (2002). Gelatinase, B., and TIMP-1 are regulated in a cell- and time-dependent manner in association with neuronal death and glial reactivity after global forebrain ischemia. Eur. J. Neurosci. 15, 19–32. 10.1046/j.0953-816x.2001.01838.x 11860503

[B94] Rodríguez J. A. Sobrino T. Orbe J. Purroy A. Martínez-Vila E. Castillo J.. (2013). proMetalloproteinase-10 is associated with brain damage and clinical outcome in acute ischemic stroke. J. Thromb. Haemost. 11, 1464–1473. 10.1111/jth.12312 23742289

[B95] Rodrigues S. F. Granger D. N. (2015). Blood cells and endothelial barrier function. Tissue Barriers 3:e978720. 10.4161/21688370.2014.978720 25838983 PMC4372023

[B96] Rodríguez-Yáñez M. Castellanos M. Blanco M. García M. M. Nombela F. Serena J.. (2006). New-onset hypertension and inflammatory response/poor outcome in acute ischemic stroke. Neurology 67, 1973–1978. 10.1212/01.wnl.0000247064.53130.91 17159103

[B97] Romanic A. M. White R. F. Arleth A. J. Ohlstein E. H. Barone F. C. (1998). Matrix metalloproteinase expression increases after cerebral focal ischemia in rats: inhibition of matrix metalloproteinase-9 reduces infarct size. Stroke 29, 1020–1030. 10.1161/01.STR.29.5.1020 9596253

[B98] Rosell A. Alvarez-Sabín J. Arenillas J. F. Rovira A. Delgado P. Fernandez-Cadenas I.. (2005). A matrix metalloproteinase protein array reveals a strong relation between MMP-9 and MMP-13 with diffusion-weighted image lesion increase in human stroke. Stroke 36, 1415–1420. 10.1161/01.STR.0000170641.01047.cc 15947272

[B99] Rosell A. Cuadrado E. Ortega-Aznar A. Hernández-Guillamon M. Lo E. H. Montaner J. (2008). MMP-9-positive neutrophil infiltration is associated to blood-brain barrier breakdown and basal lamina type IV collagen degradation during hemorrhagic transformation after human ischemic stroke. Stroke 39, 1121–1126. 10.1161/STROKEAHA.107.500868 18323498

[B100] Rosell A. Ortega-Aznar A. Alvarez-Sabín J. Fernández-Cadenas I. Ribó M. Molina C. A.. (2006). Increased brain expression of matrix metalloproteinase-9 after ischemic and hemorrhagic human stroke. Stroke 37, 1399–1406. 10.1161/01.STR.0000223001.06264.af 16690896

[B101] Rosenberg G. A. (2002). Matrix metalloproteinases in neuroinflammation. Glia 39, 279–291. 10.1002/glia.10108 12203394

[B102] Rosenberg G. A. Cunningham L. A. Wallace J. Alexander S. Estrada E. Y. Grossetete M.. (2001). Immunohistochemistry of matrix metalloproteinases in reperfusion injury to rat brain: activation of MMP-9 linked to stromelysin-1 and microglia in cell cultures. Brain Res. 893, 104–112. 10.1016/S0006-8993(00)03294-7 11222998

[B103] Rosenberg G. A. Estrada E. Y. Dencoff J. E. (1998). Matrix metalloproteinases and TIMPs are associated with blood-brain barrier opening after reperfusion in rat brain. Stroke 29, 2189–2195. 10.1161/01.STR.29.10.2189 9756602

[B104] Rosenberg G. A. Yang Y. (2007). Vasogenic edema due to tight junction disruption by matrix metalloproteinases in cerebral ischemia. Neurosurg. Focus 22, E4. 10.3171/foc.2007.22.5.5 17613235

[B105] Sandoval K. E. Witt K. A. (2008). Blood-brain barrier tight junction permeability and ischemic stroke. Neurobiol. Dis. 32, 200–219. 10.1016/j.nbd.2008.08.005 18790057

[B106] Schnoor M. Parkos C. A. (2008). Disassembly of endothelial and epithelial junctions during leukocyte transmigration. Front. Biosci. 13, 6638–6652. 10.2741/3178 18508684

[B107] Shigemori Y. Katayama Y. Mori T. Maeda T. Kawamata T. (2006). Matrix metalloproteinase-9 is associated with blood-brain barrier opening and brain edema formation after cortical contusion in rats. Acta Neurochir. Suppl. 96, 130–133. 10.1007/3-211-30714-1_29 16671440

[B108] Si-Tayeb K. Monvoisin A. Mazzocco C. Lepreux S. Decossas M. Cubel G.. (2006). Matrix metalloproteinase 3 is present in the cell nucleus and is involved in apoptosis. Am. J. Pathol. 169, 1390–1401. 10.2353/ajpath.2006.060005 17003494 PMC1780186

[B109] Sotgiu S. Zanda B. Marchetti B. Fois M. L. Arru G. Pes G. M.. (2006). Inflammatory biomarkers in blood of patients with acute brain ischemia. Eur. J. Neurol. 13, 505–513. 10.1111/j.1468-1331.2006.01280.x 16722977

[B110] Steadman R. St John P. L. Evans R. A. Thomas G. J. Davies M. Heck L. W.. (1997). Human neutrophils do not degrade major basement membrane components during chemotactic migration. Int. J. Biochem. Cell Biol. 29, 993–1004. 10.1016/S1357-2725(97)00038-1 9375379

[B111] Sternlicht M. D. Werb Z. (2001). How matrix metalloproteinases regulate cell behavior. Annu. Rev. Cell Dev. Biol. 17, 463–516. 10.1146/annurev.cellbio.17.1.463 11687497 PMC2792593

[B112] Sumii T. Lo E. H. (2002). Involvement of matrix metalloproteinase in thrombolysis-associated hemorrhagic transformation after embolic focal ischemia in rats. Stroke 33, 831–836. 10.1161/hs0302.104542 11872911

[B113] Svedin P. Hagberg H. Sävman K. Zhu C. Mallard C. (2007). Matrix metalloproteinase-9 gene knock-out protects the immature brain after cerebral hypoxia-ischemia. J. Neurosci. 27, 1511–1518. 10.1523/JNEUROSCI.4391-06.2007 17301159 PMC6673738

[B114] Tan H. K. Heywood D. Ralph G. S. Bienemann A. Baker A. H. Uney J. B. (2003). Tissue inhibitor of metalloproteinase 1 inhibits excitotoxic cell death in neurons. Mol. Cell. Neurosci. 22, 98–106. 10.1016/S1044-7431(02)00024-6 12595242

[B115] Tang Y. Xu H. Du X. Lit L. Walker W. Lu A.. (2006). Gene expression in blood changes rapidly in neutrophils and monocytes after ischemic stroke in humans: a microarray study. J. Cereb. Blood Flow Metab. 26, 1089–1102. 10.1038/sj.jcbfm.9600264 16395289

[B116] Tsuji K. Aoki T. Tejima E. Arai K. Lee S. R. Atochin D. N.. (2005). Tissue plasminogen activator promotes matrix metalloproteinase-9 upregulation after focal cerebral ischemia. Stroke 36, 1954–1959. 10.1161/01.STR.0000177517.01203.eb 16051896

[B117] Tsuruoka A. Atsumi C. Mizukami H. Imai T. Hagiwara Y. Hasegawa Y. (2014). Effects of edaravone, a free radical scavenger, on circulating levels of MMP-9 and hemorrhagic transformation in patients with intravenous thrombolysis using low-dose alteplase. J. Stroke Cerebrovasc. Dis. 23, 2894–2899. 10.1016/j.jstrokecerebrovasdis.2014.07.022 25282183

[B118] Turba M. E. Forni M. Gandini G. Gentilini F. (2007). Recruited leukocytes and local synthesis account for increased matrix metalloproteinase-9 activity in cerebrospinal fluid of dogs with central nervous system neoplasm. J. Neurooncol. 81, 123–129. 10.1007/s11060-006-9213-2 16826366

[B119] Van den Steen P. E. Dubois B. Nelissen I. Rudd P. M. Dwek R. A. Opdenakker G. (2002). Biochemistry and molecular biology of gelatinase B or matrix metalloproteinase-9 (MMP-9). Crit. Rev. Biochem. Mol. Biol. 37, 375–536. 10.1080/10409230290771546 12540195

[B120] Vandooren J. Van den Steen P. E. Opdenakker G. (2013). Biochemistry and molecular biology of gelatinase B or matrix metalloproteinase-9 (MMP-9): the next decade. Crit. Rev. Biochem. Mol. Biol. 48, 222–272. 10.3109/10409238.2013.770819 23547785

[B121] Vukasovic I. Tesija-Kuna A. Topic E. Supanc V. Demarin V. Petrovcic M. (2006). Matrix metalloproteinases and their inhibitors in different acute stroke subtypes. Clin. Chem. Lab. Med. 44, 428–434. 10.1515/cclm.2006.079 16599837

[B122] Wang C. X. Ding X. Noor R. Pegg C. He C. Shuaib A. (2009). Rosiglitazone alone or in combination with tissue plasminogen activator improves ischemic brain injury in an embolic model in rats. J. Cereb. Blood Flow Metab. 29, 1683–1694. 10.1038/jcbfm.2009.87 19553906

[B123] Wang Q. Doerschuk C. M. (2002). The signaling pathways induced by neutrophil-endothelial cell adhesion. Antioxid. Redox Signal. 4, 39–47. 10.1089/152308602753625843 11970842

[B124] Wang X. Lee S. R. Arai K. Tsuji K. Rebeck G. W. Lo E. H. (2003). Lipoprotein receptor-mediated induction of matrix metalloproteinase by tissue plasminogen activator. Nat. Med. 9, 1313–1317. 10.1038/nm926 12960961

[B125] Yan C. Boyd D. D. (2007). Regulation of matrix metalloproteinase gene expression. J. Cell. Physiol. 211, 19–26. 10.1002/jcp.20948 17167774

[B126] Yang Y. Estrada E. Y. Thompson J. F. Liu W. Rosenberg G. A. (2007). Matrix metalloproteinase-mediated disruption of tight junction proteins in cerebral vessels is reversed by synthetic matrix metalloproteinase inhibitor in focal ischemia in rat. J. Cereb. Blood Flow Metab. 27, 697–709. 10.1515/CCLM.2006.079 16850029

[B127] Yang Y. Jalal F. Y. Thompson J. F. Walker E. J. Candelario-Jalil E. Li L.. (2011). Tissue inhibitor of metalloproteinases-3 mediates the death of immature oligodendrocytes via TNF-alpha/TACE in focal cerebral ischemia in mice. J. Neuroinflammation 8:108. 10.1186/1742-2094-8-108 21871134 PMC3180275

[B128] Yushchenko M. Weber F. Mader M. Schöll U. Maliszewska M. Tumani H.. (2000). Matrix metalloproteinase-9 (MMP-9) in human cerebrospinal fluid (CSF): elevated levels are primarily related to CSF cell count. J. Neuroimmunol. 110, 244–251. 10.1016/S0165-5728(00)00339-8 11024556

[B129] Zhao B. Q. Tejima E. Lo E. H. (2007). Neurovascular proteases in brain injury, hemorrhage and remodeling after stroke. Stroke 38, 748–752. 10.1161/01.STR.0000253500.32979.d1 17261731

[B130] Zhao B. Q. Wang S. Kim H. Y. Storrie H. Rosen B. R. Mooney D. J.. (2006). Role of matrix metalloproteinases in delayed cortical responses after stroke. Nat. Med. 12, 441–445. 10.1038/nm1387 16565723

[B131] Zhao J. K. Guan F. L. Duan S. R. Zhao J. W. Sun R. H. Zhang L. M.. (2013). Effect of focal mild hypothermia on expression of MMP-9, TIMP-1, Tau-1 and beta-APP in rats with cerebral ischaemia/reperfusion injury. Brain Inj. 27, 1190–1198. 10.3109/02699052.2013.804206 23895636

[B132] Zozulya A. Weidenfeller C. Galla H. J. (2008). Pericyte-endothelial cell interaction increases MMP-9 secretion at the blood-brain barrier *in vitro*. Brain Res. 1189, 1–11. 10.1016/j.brainres.2007.10.099 18061148

